# Changes in Plasma Kynurenic Acid Concentration in Septic Shock Patients Undergoing Continuous Veno-Venous Haemofiltration

**DOI:** 10.1007/s10753-013-9733-9

**Published:** 2013-09-17

**Authors:** Wojciech Dabrowski, Tomasz Kocki, Jacek Pilat, Jolanta Parada-Turska, Manu L. N. G. Malbrain

**Affiliations:** 1 Department of Anaesthesiology and Intensive Therapy, Medical University of Lublin, Jaczewskiego Street 8, 20-954 Lublin, Poland; 2 Department of Clinical and Experimental Pharmacology, Medical University of Lublin, Lublin, Poland; 3 Department of General Surgery, Transplantology and Clinical Nutrition, Medical University of Lublin, Lublin, Poland; 4 Department of Rheumatology and Connective Tissue Diseases, Medical University of Lublin, Lublin, Poland; 5 Department of Intensive Care Unit and High Care Burn Unit, Ziekenhuis Netwek Antwerpen, ZNA Campus Stuivenberg/St-Erasmus, Antwerp, Belgium

**Keywords:** kynurenic acid, inflammatory markers, continuous veno-venous haemofiltration, septic shock, acute kidney injury

## Abstract

Kynurenic acid (KYNA) is one of the end products of tryptophan metabolism. The aim of this study was to analyse plasma KYNA concentration in septic shock patients (SSP) with acute kidney injury (AKI) undergoing continuous veno-venous haemofiltration (CVVH). Changes in KYNA content were compared to alterations in the levels of procalcitonin (PCT), C-reactive protein and lactate. Adult SSP with AKI were examined. Measurements were conducted at seven time points: before beginning CVVH and at 6, 12, 24, 48, 72 and 96 h after the beginning of CVVH. Based on clinical outcomes, the data were analysed separately for survivors and non-survivors. Twenty-seven patients were studied. CVVH was associated with reduced plasma KYNA concentration only in survivors. Plasma KYNA concentration correlated with the levels of lactate and PCT only in survivors. (1) CVVH reduced plasma KYNA concentration only in survivors; (2) lack of this reduction may predict fatal outcomes in SSP.

## BACKGROUND

The kynurenine pathway represents a major route for the peripheral metabolism of tryptophan. The process is initiated by tryptophan-2,3-dioxygenase (TDO) and indoleamine-2,3-dioxygenase (IDO). Currently, investigators focus their attention mainly on the role of IDO, which is an inducible enzyme found in cells that are involved in immune reactions [1]. It has been found that IDO is activated during bacterial or viral infection, in autoimmune disease and after severe trauma [2–4]. Circulating cytokines stimulate IDO expression and activity [4, 5]. Interestingly, increased concentrations of tryptophan metabolites, such as quinolinic acid and kynurenic acid (KYNA), depend on the severity of infection [2].

KYNA is a broad-spectrum antagonist of the ionotropic glutamate receptor and of the α7 nicotinic receptor. The role of KYNA in different brain pathologies has been widely described, whereas its peripheral function is not understood as well. The synthesis of KYNA has been documented in the heart, liver and vascular endothelium [6–8]. Importantly, kynurenine metabolites were recently implicated in the pathophysiologies of various acute and chronic diseases, including inflammation (via inhibition of T cell and natural killer function, inhibition of granulocyte activation and tumour necrosis factor alpha (TNFα) production), sepsis and trauma [2, 9–15].

Septic shock is a major cause of death in critically ill patients who are treated in intensive care units (ICUs). The mortality rate of patients in septic shock remains high and ranges between 30 and 50 % [16, 17]. It is associated with increased levels of pro-inflammatory and anti-inflammatory mediators, which cause endothelial injury and multi-organ failure. Diagnosis of the systemic inflammatory response to infection is based mainly on clinical status and biochemical markers including leukocytosis and increases in plasma levels of procalcitonin (PCT), C-reactive protein (CRP) and lactate. However, currently, there is no gold standard for the diagnosis of severe inflammatory responses. The measurement of plasma PCT concentration seems to be the most reliable diagnostic marker [18].

Acute kidney injury (AKI) is frequently associated with septic shock. Despite improvements in the treatment of AKI in septic shock patients, mortality rates remain high. Conventional daily haemodialysis is routinely used to treat AKI, but continuous veno-venous haemofiltration (CVVH) is increasingly popular [19, 20]. Slow and continuous fluid removal, cardiovascular stability and easily controlled biochemical disturbances are the main advantages of CVVH [20]. Several studies have documented significantly decreased plasma concentrations of the proinflammatory cytokines CRP and PCT during CVVH [21, 22]. Nevertheless, a decline in the levels of these mediators was not shown to be a reliable predictor of clinical outcome. Some authors have suggested that metabolites of tryptophan could be useful in the prediction of the outcomes of septic shock [9, 23–25].

It has been shown that the physiological plasma concentration of KYNA ranges between 25 and 35 nmol/L and increases to micromolar concentrations during inflammatory responses and cytokine release [26, 27]. Therefore, the aim of our study was to analyse the effect of CVVH on plasma KYNA concentrations in patients with AKI who were treated for septic shock. Moreover, changes in plasma KYNA concentrations were analysed in parallel to changes in plasma PCT, CRP and lactate levels.

## PATIENTS AND METHODS

### Ethics

This prospective observational study was conducted in the First Clinic of Intensive Therapy at the Medical University of Lublin, Poland. The study was conducted in accordance with the intensive care unit (ICU) protocol, the Declaration of Helsinki and applicable regulatory requirements as approved by the Institutional Review Board and the local institutional ethics committee. Informed consent was obtained from the legal representatives of patients because all patients were sedated and on mechanical ventilation (MV).

### Patient Selection

Adult patients treated with CVVH due to AKI related to septic shock were enrolled. Sepsis was defined as the suspicion of infection plus the systemic response to it (such as tachypnea, tachycardia and hyperthermia or hypothermia). Septic shock was defined as sepsis with evidence of altered organ perfusion associated with hypotension [28]. The diagnosis of AKI was based on RIFLE criteria [29] that included: (1) decrease in diuresis despite fluid and/or diuretic therapy to 0.3 ml/kg/h or less (*i.e.*, oliguria or anuria), (2) decrease in glomerular filtration rate (GFR) > 75 % and (3) increase in serum potassium concentration above 5.5 mmol/L combined with a threefold increase in serum creatinine level. Pregnant women, patients below the age of 18 years, patients with haematological, neoplastic, severe endocrine and metabolic disorders, transplant recipients, HIV-infected patients and those receiving immunosuppressive or steroid drugs or with a do-not-resuscitate order and life expectancy of less than one month were excluded from this study.

### Data Collection

For the entire duration of the ICU stay, relevant demographic, clinical and laboratory data were registered in an electronic database along with daily assessments of fluid balance, sepsis-related organ failure assessment (SOFA) scores, MV settings and advanced haemodynamic monitoring variables (obtained with a Swan-Ganz catheter). These data were supplemented by mortality on day 28.

The severity of illness upon ICU admission was described by the acute physiology and chronic health evaluation (APACHE-II) score. For the Glasgow coma score, the best value (before the use of sedation) was used as all the patients were sedated and intubated.

### Monitoring and Treatment Techniques

Before starting CVVH, a Swan-Ganz catheter (Arrow Int., Reading, PA, USA) was inserted via the left internal jugular vein. Its position was confirmed by chest X-ray examination. Pulmonary and systemic haemodynamic parameters were measured during the examination period. Fluid administration, vasopressor use and respiratory settings were titrated to obtain adequate oxygen saturation of haemoglobin in mixed-venous blood (S_v_O2 > 70 %) to keep the rate of oxygen delivery balanced against consumption (keeping haematocrit levels above 30 %). All patients received mineralocorticosteroid therapy (intravenous hydrocortisone infusion at a daily dose of 300 mg). Antibiotic therapy was based on bacteriological results. Systemic arterial blood pressures were measured continuously in the radial artery.

Post-dilution CVVH was performed with a Prismaflex (Gambro, AB, Lund, Sweden) CVVH monitor. A percutaneous double-lumen catheter (Arrow Int., Reading, Pennsylvania, USA) was inserted into the femoral or jugular vein using the standard Seldinger technique. Blood was extracorporeally circulated at a rate of 200 to 250 ml/min through a polysulfone membrane ST 150 (Gambro, AB, Lund, Sweden). High-volume CVVH was performed (70 to 75 ml/kg body weight) during the first 6 h of therapy, followed by low-volume CVVH (35 ml/kg body weight) throughout the remainder of the treatment. Fluid removal at a constant rate of 150 to 200 ml/h was started 6 h after the initiation of CVVH. The ultrafiltration (UF) volume was measured hourly and was balanced according to haemodynamic status. Anticoagulation was achieved using the criteria described by Ostermann and colleagues [30]. The APTT was measured every 4 h. The filter was changed after 48 h or when an excessive increase in transmembrane pressure prevented ultrafiltration.

All patients with gastrointestinal dysfunction grade III or IV [31] received total parenteral nutrition using SMOF Kabiven solution (Fresenius Kabi, Uppsala, Sweden). The mean delivery of calories ranged from 30 to 35 kcal/kg body weight per day. In patients with detectable bowel sounds, the enteral feeding was supplemented with Nutrison Multi Fibre (Nutricia, UK) to reach the target caloric energy supply per day.

### Study Protocol and Primary Endpoint

Measurements were taken before CVVH (0 h), and 6, 12, 24, 48, 72 and 96 h after the beginning of CVVH. Based on clinical outcomes, patients were allocated into two groups: patients who survived CVVH treatment (survivors) and patients who died during CVVH, but after 96 h from the beginning of CVVH (non-survivors).

### Determination of Studied Parameters

Plasma KYNA concentration was measured fluorometrically. Briefly, blood plasma was deproteinated with 50 % trichloroacetic acid and centrifuged. The supernatant was applied to cation-exchange resin (Dowex 50W+, Sigma). KYNA was eluted, subjected to HPLC (Hewlett Packard 1050 HPLC system: ESA catecholamine HR-30, 3 μm, C_18_ reverse-phase column) and quantified fluorometrically (Hewlett Packard 1046A fluorescence detector: excitation 344 nm, emission 398 nm). KYNA concentrations were expressed in nM. [32, 33].

Plasma CRP concentration was measured according to the immunoturbidimetric method with latex (ADVIA 1650 analyser, Siemens, USA). Values higher than 5 mg/L were considered clinically important. Plasma PCT concentration was measured using the electrochemiluminescence (ECLIA) method (Cobas e601 analyser, Roche, France). Values greater than 2 ng/mL were considered clinically important. Plasma lactate concentrations were measured with the Cobas b221 analyser (Roche, France). Values greater than 1 mmol/L were considered clinically significant.

## STATISTICS

Means and standard deviations (SD) were calculated for parametric data. The value at time point 0 was regarded as the baseline. Categorical variables were compared using the *χ* ^2^ and Fisher's exact test and Yates' correction was applied. Student's unpaired *t* test was used to analyse variables with normal distribution. Non-parametric data were analysed statistically using the Wilcoxon signed-rank test and the Kruskal–Wallis ANOVA test for initial detection of differences. Dunnett's post hoc and Spearman's rank correlation tests were used for inter-point and inter-group comparisons. Additionally, the Spearman's rank correlation test was used for the overall analysis. A *P* value of *P* < 0.05 was considered to be significant. A preliminary estimate of sample size was based on expected differences in plasma KYNA concentrations between baseline and 96 h. With a type I error of 0.05 and a type II error of 0.2, the required sample size was 19–23 patients. The dropout rate was estimated at 10 %; thus, a minimum of 26 patients was examined. The sample size was determined using Statistica 9 software. The power of all statistical tests was determined using G*Power software(1 − β).

## RESULTS

Forty septic shock patients with AKI were studied; of these, 12 patients were excluded: six patients died during the study period, and their data were thus incomplete; one patient required massive fluid resuscitation due to severe coagulation disorders following bleeding and died at day 5 following ICU admission; three required emergency laparotomies due to intra-abdominal bleeding (two of them died during CVVH); one patient was excluded due to very large haemolysis during sample preparation; and one was excluded because his legal representatives withdrew consent for blood collection. Therefore, this study was completed for 28 patients; 17 male and 11 female aged 60 ± 12 years (Table 1). Fourteen patients were treated for severe pneumonia due to bacterial infection (*Pseudomonas aeruginosa*, *Klebsiella pneumoniae*, *Acinetobacter baumannii*, *Streptococcus pneumoniae*, *Candida glabrata*), six for peritonitis (*Enterococcus faecalis*, *Escherichia coli*, *Candida albicans*, *Stenotrophomonas maltophilia*), three for urinary tract infection (*Staphylococcus haemolyticus*, *Escherichia coli*) and one patient was treated after hysterectomy due to pyometra (*Enterococcus faecalis*, *Candida albicans*). One patient was treated for sepsis related to an oral abscess (*Coagulase-negative staphylococcus*, *Candida albicans*), one patient was treated for a purulent spinal infection (*Acinetobacter baumannii*, *Staphylococcus aureus*) and one patient was treated for a purulent crotch infection (*Proteus mirabilis*, *Enterococcus faecalis*). The choice of antimicrobial therapy was determined after the culture and sensitivity analysis (Table 2). In all patients, it was administered intravenously.

**Table 1:** Patient Demographics and Some Laboratory Data

	Study population	Survivors	Non-survivors	*P* value
Age	60 ± 12	58 ± 13	65 ± 10	NS
Male	17	11	5	–
Female	11	7	4	–
APACHE II	24.64 ± 5.23	22.53 ± 4.06	29.11 ± 4.73	*P* < 0.05
SOFA	10.64 ± 2.56	9.47 ± 2.01	13.22 ± 1.3	*P* < 0.001
WBC (×10^3^ cell/μL)^0^	18.6 ± 9.02	17.83 ± 9.24	20.22 ± 8.85	NS
WBC (×10^3^ cell/μL)^96^	18.79 ± 13.26	14.61 ± 8.87	27.62 ± 16.95	*P* < 0.01
ALT^0^ (U/L)	99.17 ± 128.76	73.05 ± 115.17	154.33 ± 145.14	NS
ALT^96^ (U/L)	101.5 ± 139.58	45.84 ± 47.75	219 ± 194.24	*P* < 0.05
AST^0^ (U/L)	263.11 ± 613.06	291.16 ± 638.63	335.89 ± 580.14	NS
AST^96^ (U/L)	146.43 ± 243.78	56 ± 68.76	337.33 ± 359.87	*P* < 0.05
Creatinine^0^ (mg/dL)	3.49 ± 1.7	3.35 ± 1.7	3.79 ± 1.59	NS
Creatinine^96^ (mg/dL)	1.6 ± 0.85	1.38 ± 0.63	2.1 ± 1.1	*P* < 0.001
eGFR^0^ (mL/min/1.72 m^2^)	24.72 ± 8.13	24.14 ± 8.52	25.97 ± 7.8	NS
eGFR^96^ (mL/min/1.72 m^2^)	103.69 ± 48.44	134.1 ± 21.33	39.54 ± 10.58	*P* < 0.001
Dobutamine^0^	9.68 ± 3.16	9.11 ± 2.86	10.89 ± 3.72	NS
Dobutamine^96^	7.04 ± 2.17	6.33 ± 1.83	8.44 ± 2.29	*P* < 0.05
Norepinephrine^0^	0.78 ± 0.31	0.73 ± 0.29	0.97 ± 0.29	*P* < 0.05
Norepinephrine^96^	0.5 ± 0.29	0.38 ± 0.24	0.73 ± 0.27	*P* < 0.01

The mean APACHE II and SOFA scores were higher in non-survivors than in survivors. Patients who died required similar mean doses of dobutamine (microgramme per kilogramme of body weight per minute) and higher doses of norepinephrine (microgramme per kilogramme of body weight per minute) at the day of admission into ICU (Dobutamine^0^ and Norepinephrine^0^). Moreover, the doses in survivors after 96 h of CVVH were significantly higher than those in non-survivors (Dobutamine^96^ and Norepinephrine^96^). The mean quantifications of plasma WBC concentration, ALT and AST activities as well as creatinine and eGFR (estimated glomerular filtration rate) were similar in survivors and non-survivors before the start of CVVH (WBC^0^, ALT^0^ and AST^0^, Creatinine^0^ and eGFR^0^, respectively). After 96 h of CVVH, quantifications of plasma WBC, ALT and AST activities as well as Creatinine and eGFR were higher in non-survivors than in survivors (WBC^96^, ALT^96^ and AST^96^, Creatinine^96^ and eGFR^96^, respectively)

*NS*—non significant

**Table 2:** The Microbial Pathogens and Antimicrobial Therapy

	Pathogen	Antimicrobial agent
Pneumonia	*Pseudomonas aeruginosa*	Meropenem, Amikacin
	*Klebsiella pneumoniae*	Ceftazidime
	*Acinetobacter baumannii*	Meropenem
	*Streptococcus pneumoniae*	Ceftriakson
	*Candida glabrata*	Caspofungin
Peritonitis	*Enterococcus faecalis*	Vancomycin
	*Escherichia coli*	Ciprofloxacin
	*Candida albicans*	Fluconazol
	*Stenotrophomonas maltophilia*	Co-trimoxazole
Urinary tract infection	*Staphylococcus haemolyticus* (*MRCNS*)	Teicoplanin
	*Escherichia coli*	Ciprofloxacin, Amikacin
Oral abscess	*Coagulase-negative staphylococcus* (*MRCNS*)	Vancomycin
	*Candida albicans*	Fluconazol
Purulent spinal infection	*Acinetobacter baumannii*	Meropenem
	*Staphylococcus aureus* (*MRSA*)	Vancomycin
Purulent crotch infection	*Proteus mirabilis*	Ceftazidim
	*Enterococcus faecalis*	Linezolid

The mean duration of CVVH was 11.30 ± 1.67 days and was similar in both groups. Nineteen patients survived CVVH treatment (survivors group); however, one patient died at day 24 after ICU admission and two others died at days 26 and 27, respectively. Nine patients died during CVVH (32.1 %), but only after the 96-h study period (non-survivors group). At 28 days, mortality was 52.5 % for the entire group of patients enrolled in the present study.

The critical gastrointestinal dysfunction (III or IV degree) was diagnosed in all participants at the day of admission into ICU. During 96 h of treatment with CVVH, the gastrointestinal function did not improve in non-survivors, whereas the bowel sounds were detected in 74 % of survivors and the enteral feeding was supplemented at the third to fourth day of treatment.

**Fig. 1:**
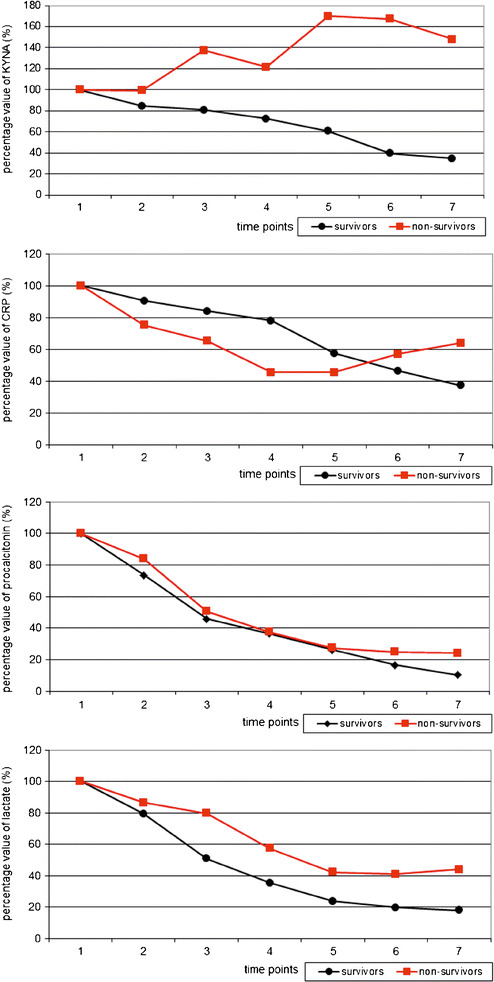
The percentage changes in plasma KYNA, procalcitonin, CRP and lactate concentrations in survivors and non-survivors. The mean of the baseline value was considered to be 100. Time points: Baseline, 6 h after the beginning of CVVH at fluid replacement rates of 70 to 75 ml/kg body weight per min without net UF, 12 h after the beginning of CVVH at fluid replacement rates of 35 ml/kg body weight per min with UF ranging between 150 and 200 ml/h, 24, 48, 72 and 96 h after the beginning of CVVH. In survivors, the percentage values of plasma KYNA concentrations decreased at 6, 12, 24, 48, 72 and 96 h (85, 81, 72, 61, 40 and 35 % of the baseline value, respectively). In non-survivors, the percentage values of plasma KYNA concentrations decreased to 99 % at 6 h of baseline value and increased at 12, 24, 48, 72 and 96 h of baseline value (138, 121, 170, 167 and 148 % of the baseline value, respectively).

The median value of plasma KYNA concentration was 73.5 nM ([14.28, 131.13]; quartiles 1 and 3, respectively) in survivors and 40.59 nM (15.73, 302.99) in non-survivors, respectively. In survivors, plasma KYNA concentrations decreased starting at 24 h of CVVH, whereas in non-survivors, they increased at 12 and 48 h of CVVH (Table 3). Concentrations did not change compared to baseline values at the remaining time points (Table 3). Plasma KYNA concentrations were significantly higher in non-survivors at 48, 72 and 96 h after the beginning of CVVH compared with those in survivors (Table 3, Fig. 1).

**Table 3:** Changes in Plasma KYNA Concentrations (in Nanomole) in Survivors and Non-Survivors

Patients	Value	Time points						
		0 h	6 h	12 h	24 h	48 h	72 h	96 h
Survivors	Quartile 1	14.28	3.76	4.92	5.12	4.2	3.34	3.94
	Median	73.5	32.55	48.58	31.53**	17.67***	22.2***	20.36***
	Quartile 3	131.13	128.62	123.71	83.8	70.2	58.02	66.71
Non-survivors	Quartile 1	15.73	28.36	43.29	50.31	42.25	37.80	47.31
	Median	40.59	51.62	51.68*	62.93	70.32*	113.24	62.21
	Quartile 3	302.99	365.17	437.96	352.43	478.65	443.40	250.52
Intergroup differences	Survivors vs non-survivors	–	–	–	–	*P* < 0.05	*P* < 0.05	*P* < 0.05

Survivors (*n* = 18) and non-survivors (*n* = 9). Time points: before CVVH (0 h), 6 h after the beginning of CVVH at fluid replacement rates of 70 to 75 ml/kg body weight per min without net UF, 12 h after the beginning of CVVH at fluid replacement rates of 35 ml/kg body weight per min with UF ranging between 150 and 200 ml/h, 24, 48, 72 and 96 h after the beginning of CVVH

*P < 0.05; **P < 0.01; ***P < 0.001 compared to the baseline value

The median value of plasma PCT concentration was significantly lower in survivors than in non-survivors at time point 0 (8.89 ng/L [6.72, 23.3] in survivors vs. 51.99 ng/L [29.55, 66.78] in non-survivors). CVVH led to decreased plasma PCT concentration in both groups; however, a higher decline was noted in survivors (Fig. 1, Table 4). Importantly, clinically significant elevation of plasma PCT concentrations was observed throughout the study in non-survivors.

**Table 4:** Changes in Plasma Procalcitonin Concentrations (in Nanogramme per Millilitre) in Survivors and Non-survivors

Patients	Value	Time points						
		0 h	6 h	12 h	24 h	48 h	72 h	96 h
Survivors	Quartile 1	6.72	3.84	2.9	2.39	1.52	0.94	0.66
	Median	8.89	5.66**	4.53***	4.5***	3.21***	1.96***	1.46***
	Quartile 3	28.3	13.82	6.93	7.57	6.27	3.55	2.43
Non-survivors	Quartile 1	29.55	23.47	13.34	7.48	5.25	4.55	4.51
	Median	51.99	33.19*	16.96*	14.31*	14.23*	10.71*	11.8*
	Quartile 3	66.78	58.13	34.73	29.98	20.08	19.4	20.27
Intergroup differences	Survivors vs. non-survivors	*P* < 0.05	*P* < 0.05	*P* < 0.01	*P* < 0.01	*P* < 0.05	*P* < 0.01	*P* < 0.001

Time points: before CVVH (0 h), 6 h after the beginning of CVVH at fluid replacement rates of 70 to 75 ml/kg body weight per min without net UF, 12 h after the beginning of CVVH at fluid replacement rates of 35 ml/kg body weight per min with UF ranging between 150 and 200 ml/h, 24, 48, 72 and 96 h after the beginning of CVVH

*P < 0.05; **P < 0.01; ***P < 0.001 compared to the baseline value

The median value of CRP prior to CVVH was 198.40 mg/L [159.95, 210.81] and 321.90 mg/L [205.03, 386.24] in survivors and non-survivors, respectively. In both groups, CVVH led to decreased plasma concentrations; however, lower values were noted in survivors after 72 h of CVVH (Fig. 1, Table 5).

**Table 5:** Changes in Plasma CRP Concentrations (in Milligramme per Litre) in Survivors and Non-survivors

Patients	Value	Time points						
		0 h	6 h	12 h	24 h	48 h	72 h	96 h
Survivors	Quartile 1	159.95	134.69	128.2	133.15	98.99	73.23	49.27
	Median	198.4	171.57**	187.3*	154.3**	118.22***	96.69***	75.6***
	Quartile 3	210.81	198.35	204.71	200.62	135.06	110.8	89.34
Non-survivors	Quartile 1	205.03	165.23	152.04	109.29	95.66	146	130.4
	Median	321.9	219.42*	230.8*	128.07*	131.6*	181.2*	203.09*
	Quartile 3	386.24	289.47	256.16	148.94	163.37	204.04	270.67
Intergroup differences	Survivors vs. non-survivors	–	–	–	–	–	*P* < 0.01	*P* < 0.01

Time points: before CVVH (0 h), 6 h after the beginning of CVVH at fluid replacement rates of 70 to 75 ml/kg body weight per min without net UF, 12 h after the beginning of CVVH at fluid replacement rates of 35 ml/kg body weight per min with UF ranging between 150 and 200 ml/h, 24, 48, 72 and 96 h after the beginning of CVVH

*P < 0.05; **P < 0.01; ***P < 0.001 compared to the baseline value

The baseline value of plasma lactate concentration was significantly lower in survivors than in non-survivors during the entire study period (5.20 mM [4, 8.4] vs. 9.45 mM [8.78, 11.23] in survivors and non-survivors, respectively). Similar to what was observed for PCT and CRP, CVVH led to decreased plasma lactate concentrations in survivors and non-survivors (Fig. 1); however, the decline was greater in survivors than in non-survivors (Table 6).

**Table 6:** Changes in Plasma Lactate Concentrations (in Millimolar) in Survivors and Non-Survivors

Patients	Value	Time points						
		0 h	6 h	12 h	24 h	48 h	72 h	96 h
Survivors	Quartile 1	4	3.3	2.2	1.7	1.25	0.95	0.65
	Median	5.2	3.6**	3***	2.1***	1.4***	1.1***	0.9***
	Quartile 3	8.4	6.6	3.75	2.55	1.75	1.2	1.25
Non-survivors	Quartile 1	8.78	7.48	5.53	4.3	3.03	3.05	3.25
	Median	9.45	7.6*	7.7	5.5*	3.9*	3.55**	3.85*
	Quartile 3	11.23	9.18	9.3	6.55	4.7	4.1	4.53
Intergroup differences	Survivors vs. non-survivors	*P* < 0.05	*P* < 0.05	*P* < 0.001	*P* < 0.001	*P* < 0.001	*P* < 0.001	*P* < 0.001

Time points: before CVVH (0 h), 6 h after the beginning of CVVH at fluid replacement rates of 70 to 75 ml/kg body weight per min without net UF, 12 h after the beginning of CVVH at fluid replacement rates of 35 ml/kg body weight per min with UF ranging between 150 and 200 ml/h, 24, 48, 72 and 96 h after the beginning of CVVH

*P < 0.05; **P < 0.01; ***P < 0.001 compared to the baseline value

There were significant correlations between KYNA and lactate concentrations in the studied population (*P* < 0.001, *r* = 0.54) as well as in survivors (*P* < 0.001, *r* = 0.5). KYNA concentration also showed a slight correlation with PCT concentration in the studied population (*P* < 0.001, *r* = 0.36) and in survivors (*P* < 0.001, *r* = 0.33). In studied population, plasma KYNA concentrations poorly correlated with plasma alanine aminotransferase (ALT) and aspartate aminotransferase (AST) (*P* < 0.001, *r* = 0.33 and *P* < 0.001, *r* = 0.3). Plasma KYNA concentration correlated with ALT (*P* < 0.001, *r* = 0.45) and AST (*P* < 0.001, *r* = 0.44) in non-survivors. There was no correlation between plasma KYNA and CRP concentrations.

Changes in some haemodynamic variables measured by Swan–Ganz catheter were presented in Table 7.

**Table 7:** Changes in Some Hemodynamic Variables and Fluid Balance in Survivors (S) and Non-Survivors (n-S)

	Group	Values	Time points						
			1	2	3	4	5	6	7
CVP (mmHg)	S	Quartile 1	5	7	9	9	9.5	10	10
		Median	7	9*	10**	10*	12*	10*	11*
		Quartile 3	11	11	12	12.5	12	12.5	13
	n-S	Quartile 1	4.5	7.5	9.3	8.3	9.8	10	12
		Median	9	9.5	10.5	10	12.5	10	12.5
		Quartile 3	15	10.8	11	13.3	15.3	10.8	14.5
	(S/n-S)		–	–	–	–	–	–	–
MPAP (mmHg)	S	Quartile 1	18.3	19.3	18.4	20.1	22.2	23.2	22.4
		Median	20.6	22	21.3	22.1	25.3*	26.6*	24.9*
		Quartile 3	24	25.5	26.6	25.3	28.1	28.4	27
	n-S	Quartile 1	17.6	19.7	18.9	20.5	21.9	22	21.3
		Median	19.6	20.8	20.3	23.2	23.3	23.1	23
		Quartile 3	35.3	27	32	25.8	33.5	25.9	34
	(S/n-S)		–	–	–	–	–	–	–
PCWP (mmHg)	S	Quartile 1	9	10	11.5	11.5	11	13	12.5
		Median	12	13	13	14	14	14	14
		Quartile 3	15	15	14.5	15.5	15	15	15
	n-S	Quartile 1	9.4	10.2	13.1	16	17.3	17	17
		Median	10	12	13.5	16	18.5	17	17.5
		Quartile 3	17.5	13.4	14	19	19	17.8	18
	(S/n-S)		–	–	–	*P* < 0.01	*P* < 0.001	*P* < 0.01	*P* < 0.001
SVI (ml/m^2^)	S	Quartile 1	27	26	33	29	32	34	29
		Median	38	36	37	36	37	41	38
		Quartile 3	43	42	45	42	42	44	43
	n-S	Quartile 1	34	32	31	28	28	25	30
		Median	35	36	44	30	43	29	33
		Quartile 3	45	36	49	33	53	43	50
	(S/n-S)		–	–	–	–	–	–	–
SVR (dyn × s × cm^−5^)	S	Quartile 1	473.5	462	504	644	645	645	654
		Median	578	563	671	754	726	733	785
		Quartile 3	918	732	722	902	860	941	970
	n-S	Quartile 1	414.3	370.3	469.5	485.7	455	618.2	570.4
		Median	606.8	517.5	486	509	495.3	675	585.2
		Quartile 3	743	717.8	487.5	514.9	608.3	807.2	822.9
	(S/n-S)		–	–	–	*P* < 0.05	*P* < 0.05	–	–
PVR (dyn × s × cm^−5^)	S	Quartile 1	69	68	60	66.1	69	89.2	91
		Median	86	84	76	114	127	125	125*
		Quartile 3	120.8	113	113.3	133.4	199.5	150.9	145
	n-S	Quartile 1	58.5	54	55	38	32	51	45
		Median	108.8	97	58	62	80	63.5	80.6
		Quartile 3	224	169	144	70	145	122	138
	(S/n-S)		–	–	–	–	–	*P* < 0.05	–
Fluid balance (mL)	S	Mean ± SD	0	2,291 ± 535	−224 ± 655	−785 ± 791	−920 ± 1,063	−696 ± 916	−967 ± 10,929
	n-S	Mean ± SD	0	2,954 ± 374	−110 ± 254	−464 ± 1,235	113 ± 315.4*	404 ± 173*	345 ± 418*
	(S/n-S)		–	*P* < 0.01	–	–	*P* < 0.01	*P* < 0.001	*P* < 0.01

Time points: before the beginning of CVVH and 6, 12, 24, 48, 72 and 96 h after the beginning of CVVH (time points 1 through 7, respectively)

*CVP* central venous pressure, *MPAP* mean pulmonary arterial pressure, *SVI* stroke volume index, *PCWP* pulmonary capillary wedge pressure, *SVR* systemic vascular resistances, *PVR* pulmonary vascular resistances

*P < 0.05; **P < 0.01; ***P < 0.001—significant differences compared to baseline

## DISCUSSION

We found that the concentration of KYNA in the plasma of septic patients with good clinical outcomes decreased gradually over the course of CVVH. Similarly, the levels of CRP, PCT and lactate decreased gradually during CVVH in this group of patients. In contrast, the concentration of KYNA in the plasma of septic patients with poor clinical results did not decrease over the course of CVVH. In fact, an increase in KYNA concentration was observed. At the same time, the concentrations of CRP, PCT and lactate decreased during CVVH in this group of patients. Plasma KYNA concentration correlated with lactate and PCT only in patients with good outcomes and with ALT and AST in patients with poor clinical outcomes. No correlations were identified between KYNA and CRP, PCT or lactate in septic patients with poor clinical outcomes.

Numerous authors have described an increase in plasma kynurenine concentration and IDO activity in sepsis and in septic shock patients [2, 9, 24, 25]. Moreover, the activity of IDO, which was calculated from the ratio of kynurenine to tryptophan, has been suggested as a marker in the diagnosis of sepsis, septic shock or other diseases. Its activity and the resulting higher levels of kynurenine have been correlated with the severity of sepsis/septic shock, and their persistently elevated values have been noted in non-survivors (*i.e.*, they increased during the initial four days in non-survivor patients) [2, 24, 25]. Tryptophan degradation was also higher after major trauma in non-survivor patients compared to survivors [9]. Some investigators have postulated that the maximum kynurenine/tryptophan ratio was detectable from 1 to 4 days after positive blood culture in septic patients, was stratified by causative bacteria and decreased on recovery. In addition, they observed the highest kynurenine/tryptophan ratios in patients with β-haemolytic streptococcal bacteraemia and the lowest ratios in those with *E*. *coli* bacteraemia. The ratio was also significantly higher in septic patients treated for pneumonia compared with those treated for urinary tract infections [23].

Kynurenine is synthesised metabolically from tryptophan. The activation of TDO and IDO following infection decreases the concentration of tryptophan in plasma and increases that of kynurenine [5, 34]. The stimulation of IDO increases tryptophan catabolism and contributes to the synthesis of the several tryptophan metabolites [11]. Kynurenine is an intermediate metabolite on the kynurenine pathway. It is mainly metabolised by two enzymes, kynurenine-3-hydroxylase and kynureninase. The resulting metabolites are 3-hydroxykynurenine, anthranilic acid, 3-hydroxyanthranilic acid, quinolinic acid and nicotinamide adenine dinucleotide (NAD^+^). It is important to note that this process progresses both *in vivo* and *in vitro*. Therefore, the precise measurement of plasma kynurenine concentrations in collected samples requires deactivation of the processes described above. In contrast to kynurenine, KYNA is not metabolised and is stable in solution. A precise and efficient method of separation has been developed using Dowex resin. Moreover, a sensitive chromatographic method has also been developed that enables the determination of KYNA concentrations in femtomolar concentrations. Therefore, measurement of KYNA content may be a good candidate for a laboratory indicator of sepsis.

The plasma KYNA concentration depends on tryptophan and KYNA supplementation, the activity of kynurenine enzymes and the rate of KYNA elimination.

Nutritional habits were strictly controlled during CVVH therapy. During the first few days, all patients received total parenteral nutrition containing 4.5 pmol/mL of KYNA (data not shown) and 1.5 g of tryptophan (Smof Kabiven). It is noteworthy that more than 90 % of plasma tryptophan is metabolised via the kynurenine pathway [35]. When abdominal sounds were detectable, enteral protein nutrition was started. The KYNA content in Nutrison Multi Fibre is 12.5 pmol/L. In all cases, the nutritional preparations were administered continuously, which maintained constant amounts of KYNA and its precursors. Interestingly, treatment with KYNA, which is *N*-methyl-d-aspartate receptor antagonist decreased obstruction-caused intestinal hypermotility in dogs and 2,4,6-trinitrobenzensulfonic acid-induced colitis in rats [14, 36]. Moreover, some experimental studies documented, that treatment with KYNA significantly reduced the level of inflammatory mediators, such as TNFα and interleukine 6 [36, 37]. On the other hand, circulating cytokines promote kynurenine metabolism and subsequent generation of tryptophan metabolites, including KYNA [2, 3, 5, 38]. Therefore, we can speculate that persistent plasma KYNA concentration results from elevated level of proinflammatory cytokines (despite CVVH) and persisted inflammation in non-survivors; however, this hypothesis requires further studies.

Endogenous KYNA is synthesised from kynurenine by kynurenine aminotransferases [13, 39]. KYNA is not further metabolised in mammals and is eliminated in urine [35]. AKI that follows septic shock may strongly determine the concentration of plasma KYNA because plasma KYNA concentration depends on the rate of its elimination through urine [27, 40, 41]. However, the rate of KYNA elimination during CVVH has not been documented. Indeed, the plasma KYNA/tryptophan ratio has been considered as a sensitive biomarker for the assessment of renal function and an increase in plasma KYNA concentration has predicted incidence of AKI [42, 43]. In patients with chronic renal diseases, increased plasma concentrations of tryptophan metabolites were reduced by renal replacement therapy [27, 42–44]. Intermittent blood purification-haemodialysis reduced plasma kynurenine and quinolinic acid by 30 and 75 %, respectively [41]. Moreover, high concentrations of both metabolites prior to haemodialysis were likely to result from the induction of TDO activity, despite the fact that the IDO activity increased rapidly in response to immune stimulation [13–15, 23, 25, 35, 41, 44]. It can be assumed that in our patients KYNA was eliminated continuously. It can also be assumed that the rate of elimination was constant and did not depend on kidney function. Thus, in the clinical situation studied here, the changes in plasma KYNA concentration depended mainly on the rate of KYNA synthesis. Therefore, a hypothesis may be proposed that the levels of KYNA fell due to its exhaustion in patients that survived septicaemia. This may indicate a beneficial effect of KYNA and should be addressed in appropriate animal models of septicaemia. Although this hypothesis does not address the reasons for the lack of decline of KYNA levels in fatal outcomes of septicaemia, it is still important to elucidate how KYNA and its analogs can benefit septic patients.

During CVVH, plasma KYNA concentrations decreased in survivor patients, whereas they were practically unchanged in non-survivor patients. It is worth stressing that other inflammatory markers, such as CRP, PCT and lactate, decreased in survivors as well as non-survivors following CVVH. Moreover, decreasing plasma KYNA concentration correlated with lactate and PCT in survivors only. It is important to note that KYNA was the only indicator for which plasma concentration was practically unchanged. Although the reason for the high content of KYNA in non-survivor patients is unknown, the prognostic value of this finding is not to be underestimated. The small sample of non-survivors significantly limited the power of statistical analysis. Therefore, our preliminary results should be verified in further studies.

## CONCLUSIONS

In conclusion, our study documented that CVVH significantly reduced plasma KYNA concentrations in septic shock patients. Surprisingly, such an effect was noted only in survivors, whereas plasma KYNA concentration was practically unchanged in non-survivors. Furthermore, decreasing plasma KYNA concentration correlated with plasma PCT and lactate concentrations. Based on our findings, we can suggest that plasma KYNA, together with CRP, PCT and lactate concentrations, may be useful as an indicator of inflammatory processes. However, larger clinical studies are necessary to demonstrate more strongly the prognostic value of KYNA in septic shock patients.
